# The Impact of COVID-19 Pandemic on Weight and Body Mass Index in Saudi Arabia: A Longitudinal Study

**DOI:** 10.3389/fpubh.2021.775022

**Published:** 2022-01-17

**Authors:** Saeed Mastour Alshahrani, Abdullah F. Alghannam, Nada Taha, Shurouq Saeed Alqahtani, Abrar Al-Mutairi, Nouf Al-Saud, Suliman Alghnam

**Affiliations:** ^1^Basic Medical Sciences Department, College of Applied Medical Sciences, King Khalid University, Abha, Saudi Arabia; ^2^Lifestyle and Health Research Center, Health Sciences Research Center, Princess Nourah Bint Abdulrahman University, Riyadh, Saudi Arabia; ^3^Population Health Department, King Abdullah International Medical Research Center, Riyadh, Saudi Arabia; ^4^Biostatistics, Epidemiology and Scientific Computing Department, King Faisal Specialist Hospital and Research Center, Riyadh, Saudi Arabia; ^5^Population Health Department, King Saud Bin Abdulaziz University for Health Sciences, Riyadh, Saudi Arabia

**Keywords:** COVID-19 pandemic, weight change, body mass index, obesity, Saudi Arabia

## Abstract

The COVID-19 pandemic has had a major impact on various health conditions. The objective of this study was to assess the impact of the COVID-19 pandemic on body weight and body mass index (BMI) in Saudi Arabia. We used electronic health records obtained from a healthcare system representing five hospitals in three different regions in the Kingdom to examine the change in weight utilizing a longitudinal design. The study included all adults who had visited outpatient clinics in two different time points, pre-2020 (years 2018 and 2019 prior to COVID-19) and post-2020 (the year 2021). Weight and BMI changes in percentages were described. Also, bivariate chi-square test, paired *t*-test, and multivariable multinomial logistic regression model were used for the analyses. A total of 165,279 individuals were included in the study. On average, a significant weight gain of 0.33 kg (95% CI: 0.29–0.36) was observed in our study. Approximately 10% of the population had shifted to either overweight or obese BMI classes during the study period, as 4.8% of those with normal BMI pre-2020 had shifted to overweight or obese classes at post-2020, and 5.1% of those who were overweight had shifted to obese class. Also, 23.1% of the population had gained 5% or more of their pre-2020 weight, while 17% had lost 5% or more. Young individuals were over three times more likely to gain 5% or more than older individuals (OR: 3.34; 95% CI: 3.12–3.56). Females had 24% higher odds to gain 5% or more of their pre-2020 weight than males (OR: 1.24; 95% CI: 1.21–1.27). Diabetics were 27% more likely to lose 5% or more than non-diabetics (OR: 1.27; 95% CI: 1.23–1.31). Our findings provide insights into the impact of COVID-19 on weight and population health. Further investment in interventions for weight management is warranted during similar circumstances such as lockdowns due to infection waves or new variants. Future studies are also needed to explore the modifications that have occurred during the pandemic in the weight-related lifestyle factors such as dietary choices and physical activity levels.

## Introduction

The prevalence of obesity has been increasing in most countries over the past five decades (1), rendering this a global phenomenon and a major public health concern. As per the WHO report, over 1.9 billion are overweight, and 650 million individuals are obese (2). The recent World Health Survey in Saudi Arabia (KSAWHS) indicated that the prevalence of overweight and obesity in 2019 was 38 and 20%, respectively, in the Kingdom (3).

On the 11^th^ of March 2020, COVID-19, an acute respiratory syndrome caused by SARS-CoV-2, was declared a global pandemic by the World Health Organization (WHO) (4). Worldwide, around 261 million cases were reported; of which, there were approximately 5.2 million deaths (5). In Saudi Arabia, approximately 550,000 cases and 8,800 deaths were documented (6). Many countries worldwide have enforced lockdowns and strict measures to reduce the spread of the virus, and they may reinforce these measures again due to new variants (7), while the world is anxiously awaiting updates on the Omicron variant (8). Saudi Arabia had imposed both partial curfews and full 24-h lockdowns between March 23-June 21, 2020, which included the holy month of Ramadan and Eid Al-Fitr (9). The measures applied to reduce the infection transmissibility include social and travel restrictions and even complete lockdowns involving a full closure of recreation centers and gyms (10).

As a result of the COVID-19 pandemic, many lifestyle habits may be unintentionally affected by lockdowns and “stay-at-home” instructions. Some important but undesirable consequences of staying at home may include weight gain, physical inactivity, and social isolation (11). The former is of particular concern, given that weight gain during adulthood is associated with a higher risk of chronic diseases (12). Further, stress and anxiety from the pandemic may be associated with health issues, including poor dietary choices and weight gain (13, 14). Several studies across the globe reported weight gain during the COVID-19 lockdowns (15–21). In the United States, two studies found that the proportion of those who have gained weight during the pandemic ranged between 22 and 27.5% (15, 16). Furthermore, results from a longitudinal study including two-time points indicated an increase of 0.62 kg from the “peak-lockdown” to “post-lockdown” periods in the United States (17). In addition, the weight gain in Europe during the pandemic ranged between 1.5 and 3 kg (18, 19). Also, an average of 0.5 kg weight increase had been observed in China (21). In Saudi Arabia, the proportion of those who reported a weight gain of 2–4 kg during the pandemic was 27.3%, with a significant increase in the proportion of those who reported “highly increased” weight during the pandemic as compared to before the pandemic (22). Such impact of the COVID-19 pandemic on weight will influence future disease burden and population health.

Risk groups of weight gain during the pandemic have been previously investigated (23–26). For example, women and youth were more likely to gain weight during the pandemic, particularly during the lockdowns (23, 24). Also, comorbidities such as hypertension and diabetes were explored for their potential association with weight change during the pandemic (25, 26). Interestingly, those with diabetes were more likely to lose weight during the pandemic, which may have been mediated by an improvement in the glycemic control (26).

Indeed, lockdowns and “stay-at-home” instructions present new obstacles to maintaining a healthy lifestyle. As of yet, the impact of the COVID-19 pandemic on weight in Saudi Arabia remains unclear. Therefore, this retrospective longitudinal study aims to compare the weight of patients visiting the National Guard Health Affairs (NGHA) in Saudi Arabia before 2020 and after 2020. The year 2020 was used as an “intervention-like” period between the two-time points (pre-2020 and post-2020), mainly because 2020 was the year in which lockdown and most restriction measures were applied in the Kingdom. This study also explores the potential risk groups associated with weight gain in our population.

## Methods

### Study Design

This is a longitudinal study based on retrospective data obtained from the patient's medical records at the National Guard Health Affairs (NGHA), Saudi Arabia. All data were retrieved from BESTcare, the hospital's electronic health system. This health system covers ~700,000 individuals receiving free full-healthcare services in five hospitals around the main three regions of Saudi Arabia (Central, Western, and Eastern). Data retrieved from the BESTCare system were grouped into two primary time points: “pre-2020,” which included data from 2018 to 2019, and “post-2020,” which included data from 2021. We identified 2020 as the intervention-like period as that it included lockdown or restriction measures. Thus, no data were collected from 2020.

### Study Population and Sample Size Calculation

The inclusion criteria were ≥17-year-old adults who visited any outpatient clinic in the health system. Weight and height measurements are routine practices for any patients visiting any of NGHA clinics taken by registered nurses. The study included measurements taken during any patient's visits in 2018, 2019, and 2021. Individuals with a history of cancer were excluded as we believe their prognosis may affect their anthropometric measurements, including weight. The final analytical sample included 165,279 subjects with two weight measurements (one during pre-2020 and another during post-2020). Assuming a mean difference of 0.2 kg between pre-and post-2020 and a standard deviation of 10 kg with a type I error of 0.05 and 80% statistical power, the sample size needed would have been 19,625 subjects (Power analysis Supplementary Table S1 in Supplementary Material); hence we believe that we have considerably sufficient power in the study with the final analytical sample of 165,279 subjects. This study was reviewed and approved by the Institutional Review Board (IRB) at King Abdullah International Medical Research center (KAIMRC).

### Measurements

Anthropometric measurements, including body weight in kilograms (kg) and height in centimeters (cm), were collected in each visit for each patient during the period of interest. Body mass index (BMI) was calculated as kg/m^2^. Based on the definition of WHO, we classified BMI classes as underweight (BMI <18.5 kg/m^2^), normal weight (BMI = 18.5–24.9 kg/m^2^), overweight (BMI = 25–29.9 kg/m^2^), and obese (BMI ≥ 30 kg/m^2^) (2). Implausible BMI values were excluded (<12 or >45).

### Covariates

Several demographic and clinical variables were retrieved, including age (17–25, 26–45, 46–64, ≥65), gender (male and female), marital status (married, unmarried, other/unknown), and geographic region (central, western, eastern). The patient's medical history of comorbidities was also obtained. We identified comorbidities including diabetes, hypertension, dyslipidemia, stroke, and COVID-19 infection based on the International Statistical Classification of Diseases and Related Health Problems (ICD-10) code (27).

### Statistical Analysis

Descriptive statistics were reported as frequency and percentages. Bivariate chi-square test was used to measure the association between several variables and percent weight change. The percent weight change was calculated by subtracting the weight at post-2020 from the weight at pre-2020 then dividing the difference by the weight at pre-2020. The change was then categorized as follows: weight gain (≥+5%), unchanged weight (< +5% and >-5%), and weight loss ( ≤ -5%). The mean difference between average weight at pre-2020 and average weight at post-2020 was tested using a paired *t*-test. In addition, a multivariable multinomial logistic regression model was used to evaluate predictors associated with the percent weight change between pre-and post-2020. The multivariable logistic regression model included the following covariates: baseline age (>65 as the reference), gender (males as the reference), marital status (unmarried/unknown individuals as the reference), geographic region (central as the reference), and medical history including diabetes, hypertension, and dyslipidemia. Model assumptions of paired *t*-test and multinomial logistic regression were evaluated. All analyses were conducted using Stata software for statistical analysis version 15 (STATA Corp., College Station, TX).

## Results

A total of 165,279 subjects were included in the study; of which, 43.2% aged 26–45, 61.3% were females, 68.8% were married, 57.2% resided in the Central region, 33% were diabetics, 26% were hypertensive, 34.3% had dyslipidemia, 1.7% had a history of stroke, and 5.3% had a previous COVID-19 infection (Table 1). The characteristics of the study participants included in the study based on their weight change status were also reported in Table 1. Those who gained 5% or more of their pre-2020 weight at post-2020 were likely to be 26–45 years old (54.5%), females (65.6%), married (61.5%), and residents of the central region (55.8%). As for comorbidities, individuals without diabetes, hypertension, or dyslipidemia had the highest percentages of weight gain by 5% or more of their pre-2020 weight with 79.3, 85.3, and 80.6%, respectively. Patients who have been diagnosed with stroke and COVID-19 had the lowest proportions of demonstrating weight gain with 1.2 and 5%, respectively (Table 1).

**Table 1:** Characteristics of the study population based on the weight change status (*N* = 165,279)^a^.

**Variable**	**Level**	**Total**	**≥5% Weight loss**	**<5% change**	**≥5% Weight gain**	***P*** **-value^b^**
Age groups, *n* (%)	17–25	23,429 (14.2)	4,160 (14.8)	10,741 (10.9)	8,528 (22.4)	<0.001
	26–45	71,328 (43.2)	12,200 (43.3)	38,373 (38.8)	20,755 (54.5)	
	46–64	48,734 (29.5)	7,783 (27.6)	34,680 (35.0)	6,271 (16.5)	
	≥65	21,788 (13.2)	4,016 (14.3)	15,223 (15.4)	2,549 (6.7)	
Gender, *n* (%)	Female	101,320 (61.3)	18,030 (64.0)	58,301 (58.9)	24,989 (65.6)	<0.001
	Male	63,959 (38.7)	10,129 (36.0)	40,716 (41.1)	13,114 (34.4)	
Marital status, *n* (%)	Married	113,693 (68.8)	19,626 (69.7)	70,631 (71.3)	23,436 (61.5)	<0.001
	Unmarried	41,606 (25.2)	6,957 (24.7)	21,779 (22.0)	12,870 (33.8)	
	Other/Unknown	9,980 (6.0)	1,576 (5.6)	6,607 (6.7)	1,797 (4.7)	
Geographic region, *n* (%)	Central	94,558 (57.2)	16,389 (58.2)	56,926 (57.5)	21,243 (55.8)	<0.001
	Western	35,306 (21.4)	5,842 (20.8)	20,811 (21.0)	8,653 (22.7)	
	Eastern	35,415 (21.4)	5,928 (21.1)	21,280 (21.5)	8,207 (21.5)	
Diabetes, *n* (%)	Yes	54,545 (33.0)	10,170 (36.1)	36,487 (36.9)	7,888 (20.7)	<0.001
	No	110,734 (67.0)	17,989 (63.9)	62,530 (63.2)	30,215 (79.3)	
Hypertension, *n* (%)	Yes	42,967 (26.0)	7,492 (26.6)	29,886 (30.2)	5,589 (14.7)	<0.001
	No	122,312 (74.0)	20,667 (73.4)	69,131 (69.8)	32,514 (85.3)	
Dyslipidemia, *n* (%)	Yes	56,642 (34.3)	9,362 (33.3)	39,897 (40.3)	7,383 (19.4)	<0.001
	No	108,637 (65.7)	18,797 (66.8)	59,120 (59.7)	30,720 (80.6)	
Stroke, *n* (%)	Yes	2,807 (1.7)	671 (2.4)	1,683 (1.7)	453 (1.2)	<0.001
	No	162,472 (98.3)	27,488 (97.6)	97,334 (98.3)	37,650 (98.8)	
COVID infection, *n* (%)	Yes	8,707 (5.3)	1,556 (5.5)	5,256 (5.3)	1,895 (5.0)	0.005
	No	156,572 (94.7)	26,603 (94.5)	93,761 (94.7)	36,208 (95.0)	

a *Data are presented as frequency and percentages (%)*.

b *Derived from Chi-square test*.

The BMI and weight changes from pre-2020 to post-2020 (Table 2) showed an average increase of 0.14 kg/m^2^ in the BMI (95% CI: 0.12–0.15) and an average increase of 0.33 kg in weight (95% CI: 0.29–0.36). Across gender, weight increased by an average of 0.19 kg (95% CI: 0.13, 0.25) among males, and 0.41 kg (95% CI: 0.37, 0.46) among females. During the study period, those with normal BMI had gained an average of 2.01 kg (95% CI: 1.94–2.07), while overweight individuals had gained an average of 0.7 kg (95% CI: 0.64–0.75). In contrast, obese individuals had lost an average of 0.93 kg (95% CI:−0.99,−0.88) (Table 2).

**Table 2:** Change in BMI and weight pre- and post-2020^a^.

		**Mean**	**S.D.^b^**	**95% C.I**.	
**BMI change (kg/m^2^)**	Post-2020	29.74	6.8	29.71	29.78
	Pre-2020	29.60	6.9	29.57	29.64
	Diff	0.14	2.9	0.12	0.15
**Weight change (kg)**					
Overall	Post-2020	76.67	18.1	76.58	76.76
	Pre-2020	76.34	18.4	76.25	76.42
	Diff	0.33	7.4	0.29	0.36
Male					
	Post-2020	80.91	18.4	80.77	81.06
	Pre-2020	80.72	18.9	80.57	80.86
	Diff	0.19	7.9	0.13	0.25
Female					
	Post-2020	73.99	17.4	73.88	74.09
	Pre-2020	73.57	17.6	73.46	73.68
	Diff	0.41	7.1	0.37	0.46
**Weight change within BMI classes (kg)**					
Normal BMI	Post-2020	60.75	10.1	60.64	60.85
	Pre-2020	58.74	8.3	58.65	58.82
	Diff	2.01	6.3	1.94	2.07
Overweight	Post-2020	72.99	10.3	72.90	73.09
	Pre-2020	72.29	8.8	72.21	72.37
	Diff	0.70	6.1	0.64	0.75
Obese	Post-2020	89.33	15.7	89.21	89.44
	Pre-2020	90.27	14.9	90.16	90.37
	Diff	−0.93	7.8	−0.99	−0.88

a *Derived from paired t-test to test for the mean difference from pre-2020 to post-2020*.

b *Standard Deviation*.

**Figure 1:**
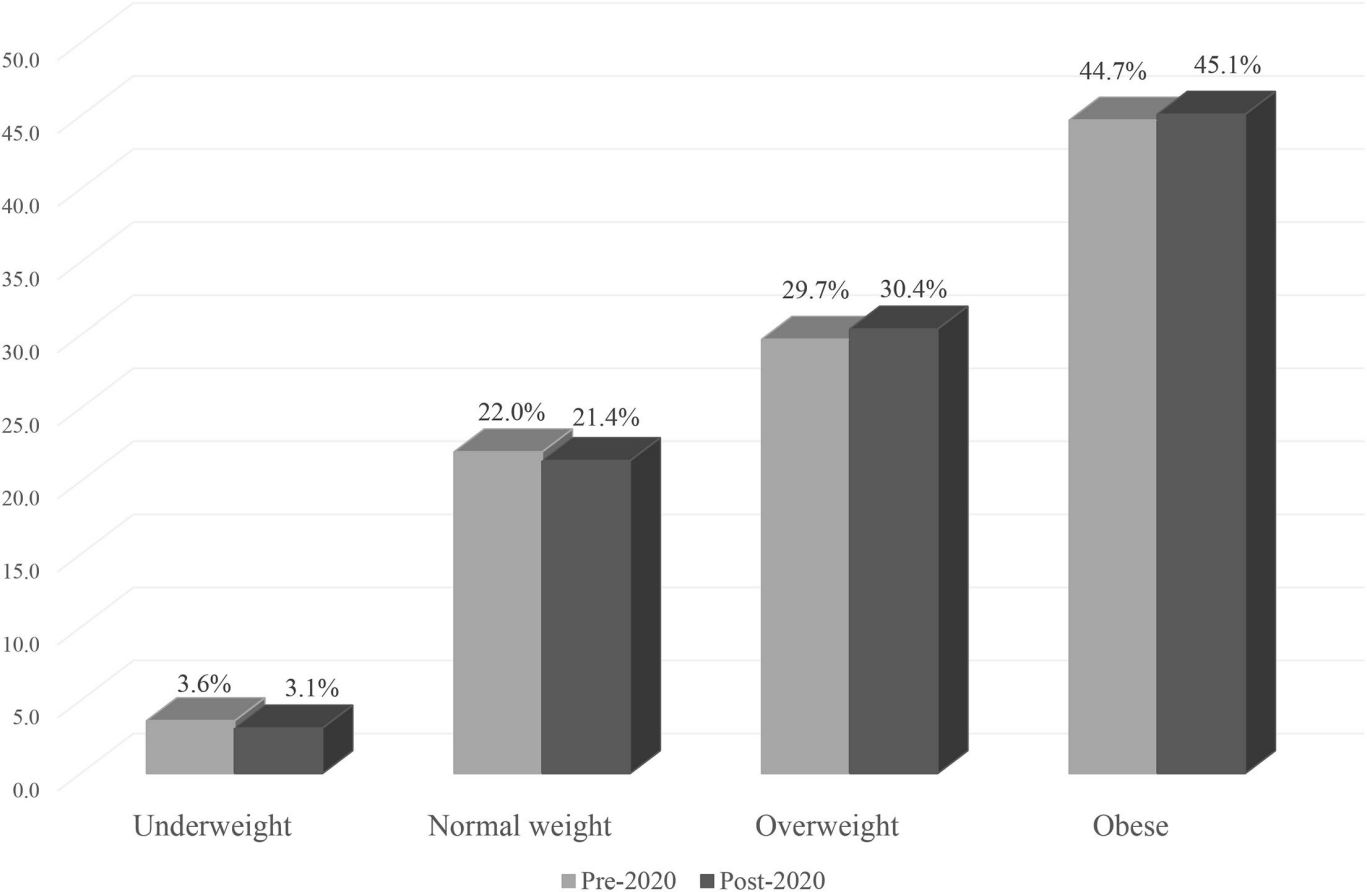
Changes in BMI classifications from pre-2020 to post-2020.

There were also increases from pre-2020 to post-2020 in the proportions of those who were overweight (29.7 vs. 30.4%) and obese (44.7 vs. 45.1%). In contrast, there was a decrease in the proportion of those who had a normal BMI pre-2020 as compared to post-2020 (22 vs. 21.4%) (Figure 1).

**Figure 2:**
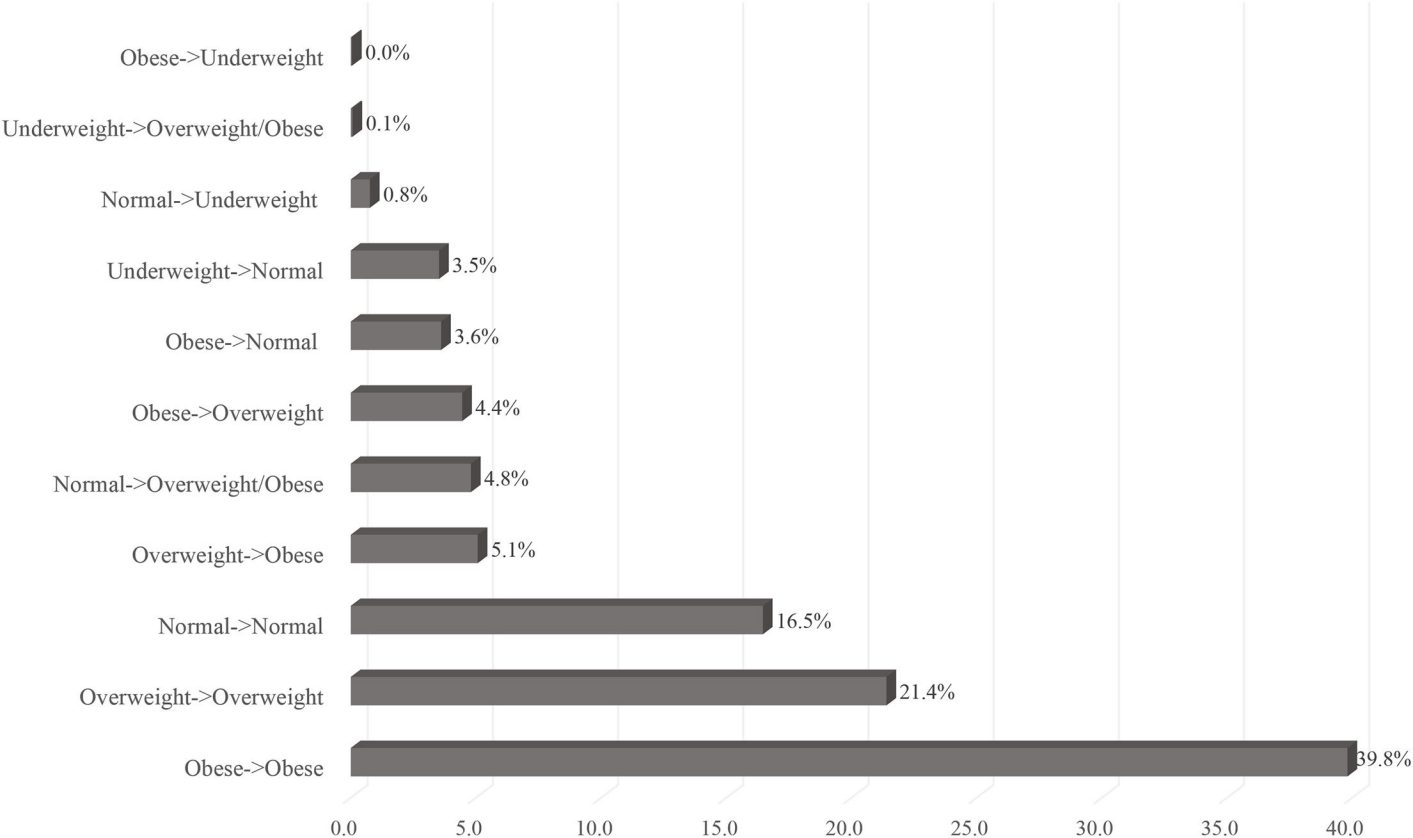
Shift in BMI classifications from pre-2020 to post-2020.

**Figure 3:**
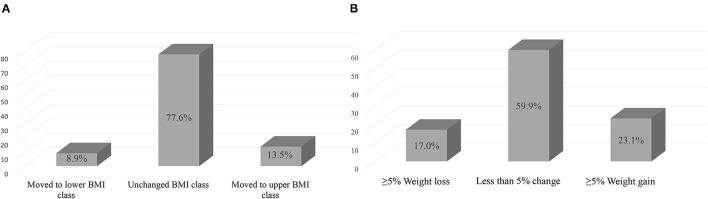
Changes in **(A)** BMI classification and **(B)** weight from pre-2020 to post-2020.

Approximately 10% of the population had shifted to either overweight or obese classes during the study period, as 4.8% of those with normal BMI pre-2020 had shifted to overweight or obese classes at post-2020, and 5.1% of those who were overweight had shifted to obese class (Figure 2). Comparing the change in BMI class between pre-2020 and post-2020, there were 13.5% who had shifted to upper BMI class (including those who were underweight to normal weight) during the study period and 8.9% who had shifted to lower BMI class, while 77.6% remained in their same pre-2020 BMI class (Figure 3A). Similarly for the change in weight, there were 23.1% had gained 5% or more of their pre-2020 weight at post-2020, and 17% had lost 5% or more of their pre-2020 weight, while around 60% had <5% or no change in their pre-2020 weight at post-2020 (Figure 3B).

Results from the multinomial logistic regression model suggested that youth (17–25) were over three times more likely to gain 5% or more of their pre-2020 weight during 2020 than the elderly (>65 years) (OR: 3.34; 95% CI: 3.12–3.56). Moreover, those who aged 26–45 years were over two-fold more likely to gain 5% or more of their pre-2020 weight during 2020 than the elderly (>65 years) (OR: 2.37; 95% CI: 2.24–2.50). Furthermore, females had 24% higher odds of gaining 5% or more of their pre-2020 weight during 2020 than males (OR: 1.24; 95% CI: 1.21–1.27). On the other hand, diabetic individuals were 27% more likely to lose 5% or more of their pre-2020 weight during 2020 than non-diabetics (OR: 1.27; 95% CI: 1.23–1.31) (Table 3).

**Table 3:** Multinomial logistic regression of predictors of 5% weight change among the study population^a^.

**Variable**		**≥5% Weight loss vs. <5% change^b^**	**≥5% Weight gain vs. <5% change^b^**
Age group	17–25	1.52 (1.42–1.63)	3.34 (3.12–3.56)
	26–45	1.23 (1.18–1.30)	2.37 (2.24–2.50)
	46–64	0.86 (0.82–0.90)	1.00 (0.94–1.05)
	>65	Ref	Ref
Gender	Female	1.24 (1.20 (1.27)	1.24 (1.21–1.27)
	Male	Ref	Ref
Marital	Married	1.07 (1.03–1.12)	1.07 (1.03–1.10)
	Unmarried/Other/Unknown	Ref	Ref
Region	Western	0.95 (0.91–0.98)	1.03 (0.99–1.06)
	Eastern	0.96 (0.92–0.99)	0.96 (0.93–0.99)
	Central	Ref	Ref
Diabetes	Yes	1.27 (1.23–1.31)	0.91 (0.88–0.94)
	No	Ref	Ref
Hypertension	Yes	1.02 (0.98–1.06)	0.95 (0.91–0.99)
	No	Ref	Ref
Dyslipidemia	Yes	0.79 (0.77–0.82)	0.64 (0.62–0.66)
	No	Ref	Ref

a *Data are presented as Odds Ratio (OR) and 95% Confidence Interval (CI)*.

b *Change of <5% is the reference category for the dependent variable (weight change)*.

## Discussion

This present study found that close to a quarter (23%) of our population have gained 5% or more of their pre-2020 weight. The average weight gain from pre-2020 to post-2020 was higher for females. These findings underline the negative externalities of COVID-19 in terms of its impact not only on infections but to other health conditions that impact population health.

Our findings are consistent with previous findings from different regions worldwide (15–21). In the United States, Zachary et al. (15) and Flanagan et al. (16) found that approximately 22 and 27.5%, respectively, of their samples had gained weight during the lockdown, which are comparable to our findings. Although their study was cross-sectional using a survey, Zachary et al. (15) collected data on an ordinal scale of multiple intervals of weight change, which may have reduced the amount of information error. Flanagan et al. (16) have used a validated instrument to collect their data, which may have enhanced the internal validity of their results. However, women were overrepresented in their study (80%); hence, the weight gain may have been slightly overreported. Further, Bhutani et al. (17) conducted a longitudinal study on weight change from the “peak-lockdown” to “post-lockdown” periods in the United States, in which they found an increase of 0.62 kg. This is relevant to our study because we also used longitudinal data from two-time points, “pre-2020 and post-2020.” However, the average weight gain difference between Bhutani et al. study and ours may be explained by the timing of data collection. They collected their baseline data during the lockdown period, while we used retrospective data from pre-2020 as baseline data. Besides, they used self-report data, which may have overestimated the weight gain in their study.

In Europe, Sidor et al. (18) reported that 30% of their sample had gained 3 kg during the COVID-19 quarantine in Poland. Furthermore, Pellegrini et al. (19) reported a 1.5 kg increase in their population average weight 1 month after the lockdown period in Italy These increases were larger than what we found in our study, which can be attributed either to the periods of their data collection (e.g., during or soon after the lockdown periods) or to a potential measurement error due to self-report weight information. In our study, on the other hand, we used longitudinal data from two time points, and the measurements were taken by registered nurses in the NGHA clinics, which should have minimized the amount of measurement error. Also in Europe, Micheletti Cremasco et al. (20) found an average weight gain of 0.4 kg which is relatively comparable to our finding. Although they collected their data soon after the lockdown, their study was cross-sectional, relying on the individual's perception of gaining weight, which could have been prone to a measurement error.

In China, Zhu et al. (21) conducted a cross-sectional study early during the pandemic and found an average gain weight of 0.5 kg, which was slightly higher than that in our study. Although they gathered a broad list of related dietary and lifestyle factors, the cross-sectional design accompanied by the self-report information may have overestimated the weight gain.

In Saudi Arabia, Abdulsalam et al. (22) found a significant increase in the proportion of those who reported “highly increased” weight during the pandemic as compared to before the pandemic (23.5 vs. 12.3%). They also found that the proportion of those who reported a weight gain of 2–4 kg during the pandemic was 27.3%, which is considerably higher than that in our study. This can be attributed to the large proportion of those aged 19–29 years in their sample (55%), whereas this age group represents roughly around 15% in our study. Nonetheless, this age group in our study has over three-fold higher odds of gaining 5% or more of their pre-2020 weight as compared to the elderly (>65 years old), which could be relevant to Abdulsalam et al. (22) findings.

Multiple factors may have led to weight gain during the pandemic such as physical inactivity, sedentary behaviors, and screen time (28–30). Access to physical activity resources, including recreation places, gyms, and sports, have been limited (31). The positive association of physical inactivity and sedentary lifestyle with weight has been well established (32). In addition, dietary habits could be another potential factor contributing to weight gain during the pandemic (28). Adverse changes in eating habits such as overeating, additional meals, snacking, and sweets have been observed during the pandemic (19, 23). Furthermore, it has been reported that individuals consumed less fruits and vegetables and more canned food during lockdown periods (15, 18).

In Saudi Arabia, the proportion of people who spent 6 h or more a day watching TV or working on computers before the pandemic had significantly increased during the pandemic as compared to before the pandemic (36.2 vs. 12.5%) (22). Further, the proportion of individuals with poor dietary habits had significantly increased during the pandemic as compared to before the pandemic (27.3 vs. 17.6%) (22). Regarding the level of physical activity in the Kingdom, the proportion of those who engaged in 3–4 h/week of physical activity before the pandemic had significantly dropped during the pandemic (16.9 vs. 11.9%) (22). The lockdown period in Saudi Arabia was imposed on March 23, 2020, and lasted until June 21, 2020, which included the holy month of Ramadan, followed by the Eid Al-Fitr holiday. The consumption of high-caloric foods enriched with sweets and salts resulting in weight gain has been observed during the holy month of Ramadan in Saudi Arabia (33). Moreover, weight gain has been previously investigated during holidays, as Yanovski et al. (34) assessed weight change during the holiday season (November-January). They found an average weight gain of 0.37 kg during the holiday seaso. This finding is very similar to our study, which the average weight gain was 0.33 kg.

Another factor that may have played a role in weight change during the pandemic is the impact on mental health. This may have contributed to or even exacerbated poor eating habits and weight gain, as reported by several studies (35–37). Levels of stress, anxiety, depression, and other mental disorders have been increased during the COVID-19 pandemic (14). Mason et al. (13) reported that eating to cope with stress is common among young adults explaining the higher likelihood of gaining weight among youth in our study. These dietary choices—triggered by mental stressors—combined with physical inactivity and sedentary lifestyle may have contributed to weight gain in our study.

Our study also found that females have higher odds of gaining weight. This may be explained by women's response to stress by poor dietary behaviors, as Tourkmani et al. (38) suggested. Additionally, we found that people with diabetes have higher odds of losing weight in our study. A possible explanation may be related to the teleconsultation offered to people with diabetes to reduce COVID-19 associated risks. A study conducted in Saudi Arabia found that telemedicine during the pandemic had improved glycemic control among those with uncontrolled type 2 diabetes (38). Improved glycemic control during the pandemic has also been reported by other studies (26, 39). Such improvement may be accompanied by other lifestyle components such as a healthy diet and physical activity.

On the other hand, we found that 17% in our study have lost 5% or more of their pre-2020 weight. That estimate is comparable to findings previously reported by other research groups (15, 17, 18). In particular, Zachary et al. (15) found approximately 19% of their participants had lost weight during the “self-quarantine,” which is also relatively comparable to our study as we found 17% of the population who had lost 5% or more of their pre-2020 weight. This could perhaps be related to those who benefited from the working at home policy to increase their physical activity levels and/or prepare their healthy meals.

Interestingly, we observed approximately 2 kg weight loss among obese individuals in our study, whereas Flanagan et al. (16) found that the percentage of obese individuals who had gained weight during the lockdown was higher than that of those with normal weight (33 vs. 27%) (16). Such a discrepancy between Flanagan et al. study (16) and ours may be attributed to the time frame of the data collection. That is, Flanagan et al. (16) collected their data using a survey during the lockdown period in the United States, while we used longitudinal data—before 2020 and after 2020— retrieved from medical records. Furthermore, the lockdown period in Saudi Arabia was lifted on June 21, 2020 (6 months before the post-2020 data was collected), which perhaps was an opportunity for obese individuals in our study to lose more weight after the lockdown.

In this present study, we used longitudinal data collected from the patient's charts. Such type of data may ensure higher validity and reliability as compared to self-reported data using surveys. However, our study has some limitations. First, the data represents those who visited the hospital in 2021. Therefore, we are unable to generalize the findings to the underlying population. However, measuring weight and height is a standard procedure in all routine appointments; hence, we have no reason to believe that our population differs substantially from the Saudi population. Second, we did not collect data on dietary behaviors or physical activity, nor did we assess the psychological disorders of the population during the pandemic. Finally, the lockdown period represented only 3 months of 2020; hence, we may have missed the potential weight fluctuations during the rest of the year. However, we believe that the lockdown effect would have resulted in more weight gain if we had assessed the weight change only during the lockdown period. Also, even though the lockdown period was only for 3 months, many organizations had continued the working-from-home policy, and many other recreation centers and gyms had remained closed. Consequently, the level of physical activity may have been similar to the lockdown period.

## Implications

Obesity is a major public health issue and associated with multiple chronic diseases and a considerable economic burden (40, 41). According to the World Health Survey in Saudi Arabia (KSAWHS), the prevalence of overweight and obesity in 2019 in the Kingdom were 38% and 20%, respectively (42). Given that the Saudi population is approximately 34 million according to the Saudi General Authority for Statistics (3), these percentages can be translated as there are approximately 13 million overweight and 7 million obese individuals in the country. Hence, if we add the increased percentage of those who were overweight pre-2020 and shifted into the obese class (5.1%) during the pandemic in our study to the current percentage in the country, it means that there would be approximately additional 357,000 obese individuals post-2020. This burden would ultimately contribute to the existing health and economic burden of obesity in Saudi Arabia.

## Conclusion

Our study provides insights into the effect of the COVID-19 pandemic on weight and obesity in Saudi Arabia. Utilizing a longitudinal design with data retrieved from medical records, we estimated the weight change from before 2020 to after 2020. We found that people tended to gain weight during the pandemic, which will negatively impact population health. Proper interventions should be considered during similar circumstances—such as lockdown due to infection waves or new variants—especially for those at higher risk of complications due to obesity. Future studies should shed light on the most relevant factors associated with weight gain during the pandemic, including dietary choices and physical activity levels.

## Data Availability Statement

The datasets presented in this article are not readily available because it must be requested from the corresponding author and they may be available upon reasonable request. Requests to access the datasets should be directed to Suliman Alghnam, ghnams@ngha.med.sa.

## Ethics Statement

The studies involving human participants were reviewed and approved by the Institutional Review Board (IRB) at King Abdullah International Medical Research Center (KAIMRC). Written informed consent from the participants' legal guardian/next of kin was not required to participate in this study in accordance with the national legislation and the institutional requirements.

## Author Contributions

SA and SMA conceived the research question and study design. SA and NT facilitated data collection. SA analyzed the data. SMA, AFA, NT, SSA, and AA-M wrote the original draft of the manuscript. SMA, NA-S, and SA reviewed and edited the manuscript. All authors contributed to the article and approved the submitted version.

## Conflict of Interest

The authors declare that the research was conducted in the absence of any commercial or financial relationships that could be construed as a potential conflict of interest.

## Publisher's Note

All claims expressed in this article are solely those of the authors and do not necessarily represent those of their affiliated organizations, or those of the publisher, the editors and the reviewers. Any product that may be evaluated in this article, or claim that may be made by its manufacturer, is not guaranteed or endorsed by the publisher.
