# Supplementary material for: The Impact of COVID-19 Pandemic on Weight and Body Mass Index in Saudi Arabia: A Longitudinal Study
Source: Front Public Health. 2022 Jan 17;9:775022. doi: 10.3389/fpubh.2021.775022 (PMC8801912; doi:10.3389/fpubh.2021.775022)
Supplement: Supplementary file 1 [file Table_1.docx]

| **Supplementary Material Table S1 Power analysis of the study entitled “The Impact of COVID-19 Pandemic on Weight and Body Mass Index in Saudi Arabia: A Longitudinal Study”, Alshahrani SM, et al.** | | | | |
| --- | --- | --- | --- | --- |
| Several Paired Mean Differences in weight between pre-2020 and post-2020 in Kilograms (kg) | Sample sized needed for several Standard Deviation of the Paired Differences | | | |
|  | SD: 5 | SD: 10 | SD: 15 | SD: 20 |
| Mean: 0.2 | 4908 | 19625 | 44152 | 78491 |
| Mean: 0.4 | 1229 | 4908 | 11040 | 19625 |
| Mean: 0.6 | 548 | 2183 | 4908 | 8723 |
| Mean: 0.8 | 309 | 1229 | 2762 | 4908 |
| Mean: 1 | 199 | 787 | 1768 | 3142 |
